# Paving the Way for Personalized Medicine in First Kidney Transplantation: Interest of a Creatininemia Latent Class Analysis in Early Post-transplantation

**DOI:** 10.3389/ti.2023.10685

**Published:** 2023-02-16

**Authors:** Héloïse Ducousso, Maxime Vallée, Thomas Kerforne, Ines Castilla, Fabien Duthe, Pierre-Jean Saulnier, Stéphanie Ragot, Antoine Thierry

**Affiliations:** ^1^ Department of Urology, University of Poitiers, CHU Poitiers, Poitiers, France; ^2^ Department of Intensive Care, University of Poitiers, CHU Poitiers, Poitiers, France; ^3^ Clinical Investigation Centre CIC1402, Poitiers University, Institut National de la santé et de la recherche médicale (INSERM), CHU Poitiers, Poitiers, France; ^4^ Department of Nephrology, Dialysis and Transplantation, University of Poitiers, CHU Poitiers, Poitiers, France

**Keywords:** donation after brain death, chronic kidney disease, latent class mixed model, kydney transplantation, trajectories, creatinine

## Abstract

Plasma creatinine is a marker of interest in renal transplantation but data on its kinetics in the first days following transplantation are scarce. The aim of this study was to identify clinically relevant subgroups of creatinine trajectories following renal transplantation and to test their association with graft outcome. Among 496 patients with a first kidney transplant included in the French ASTRE cohort at the Poitiers University hospital, 435 patients from donation after brain death were considered in a latent class modeling. Four distinct classes of creatinine trajectories were identified: “*poor recovery*” (6% of patients), “*intermediate recovery*” (47%), “good recovery” (10%) and “*optimal recovery*” (37%). Cold ischemia time was significantly lower in the “*optimal recovery*” class. Delayed graft function was more frequent and the number of hemodialysis sessions was higher in the “*poor recovery*” class. Incidence of graft loss was significantly lower in “*optimal recovery*” patients with an adjusted risk of graft loss 2.42 and 4.06 times higher in “*intermediate recovery*” and “*poor recovery*” patients, respectively. Our study highlights substantial heterogeneity in creatinine trajectories following renal transplantation that may help to identify patients who are more likely to experience a graft loss.

## Graphical Abstract

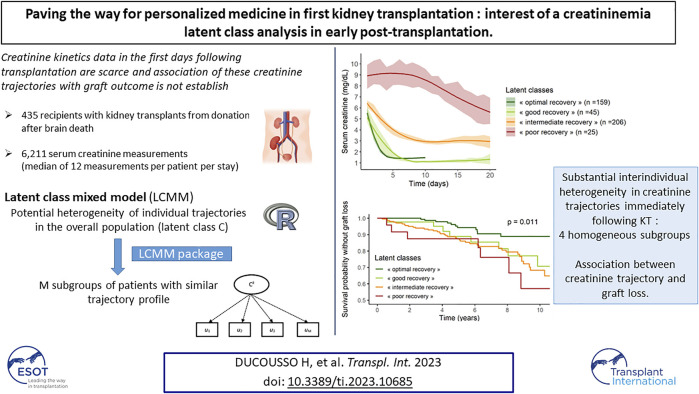

## Introduction

Kidney transplantation (KT) is optimal treatment for end-stage renal disease (ESRD), whether for the patient’s survival, his quality of life or the cost of treatment (1,2,3).

Over the last two decades, the number of KTs has increased worldwide, in the number of transplantations from both living donors (4) and cadaveric donors (5,6). This phenomenon is due to an increase of patients with kidney failure, largely linked to longer life expectancy, and to an increase in prevalence of type 2 diabetes (7). As a result, there has been a situation of kidney transplant shortage for several years which led to a rise in the number of patients on KT waiting list (6).

Reducing the need for repeated transplantation by avoiding graft loss (GL) is a major objective. Despite progress in post-transplant management, particularly in terms of immunosuppressive treatments (8,9,10), long-term graft survival has only slightly improved over recent years (10,11), with a median estimated at 14 years in France (7).

Many predictive factors of long-term graft survival have been identified in recent years such as ischemia-reperfusion syndrome (12,13), delayed graft function (DGF) (14,15). Currently, post-transplant recovery assessment of the graft is based on consensual but not very reliable parameters. Plasma creatinine is a marker of interest for the clinician, but data on its kinetics in the first days following KT are scarce. Nadir creatinine and time to reach a creatinine level below 2.83 mg/dL during the transplant patient’s hospital stay are parameters commonly used by clinicians since they are considered to be independent prognostic factors for graft survival.

Daily creatinine values during the transplant patient’s hospital stay are available and consideration of their dynamic over time could yield additional predictive information.

Many statistical models have been developed to study the evolution of quantitative markers over time. One of them is the latent class mixed model (LCMM), which is an extension of the standard linear mixed model (16). The model computes the heterogeneity of individual trajectories and identifies subgroups of patients with similar trajectory profiles, independently of their observed characteristics. This method has shown its interest in identifying subgroups of trajectories of renal function in a cohort of patients with chronic kidney disease (CKD) (17) and, more recently, in diabetic patients (18,19). While the LCMM has been applied for repeated measures of creatinine or glomerular filtration rate (GFR) (20), it has never been used to describe subgroups of trajectories of renal recovery in the days following transplantation in renal recipients. LCMM could reveal unknown heterogeneity in post-KT trajectories of renal function which could help identify kidney recipients at high risk of GL.

Therefore, our aims were 1) to evaluate the ability of the LCMM to identify subgroups of trajectories of creatinine immediately following renal transplantation, 2) to identify the clinical factors associated with the different trajectories and 3) to test the association between subgroups of trajectories and graft outcomes.

## Materials and Methods

### Study Population

Patients were included if they met the following criteria: age ≥18 years; first KT from donation after brain death (DBD) between January 2008 and July 2017 at the Poitiers university hospital and enrollment in the French ASTRE cohort (21). Non-inclusion criteria were living donors, donors after cardiac death (DCD), retransplantation, early graft failure, defined by recipient death or GL within 3 months, or primary non-function.

All patients signed informed consent before inclusion. The study follows the STROBE statement and was conducted following the principles of the Declaration of Helsinki and approved by the CNIL (Authorization number DR-2012- 518 [ps2]).

Expanded criteria donors (ECD) were defined by age >60 years or by age between 50 and 59 years with the association of two comorbidities: hypertension, creatinine ≥1.511 mg/dL or a cerebrovascular death (22,23,24,25).

### Clinical and biological data

The demographic, clinical and biological data of the recipients and donors were collected in the ASTRE database. Post-KT hemodialysis session follow-up data was obtained from the electronic health record system by the Department of Medical Information of Poitiers University Hospital. Serum creatinine was determined daily, by enzymatic method in the Biochemistry department of Poitiers University Hospital. To establish the renal function trajectories following transplantation, we considered all the creatinine determinations during the hospital stay from 24 h after the transplant to hospital discharge.

### Follow-Up and Outcome

Patients were followed up as were all the patients included in the French ASTRE cohort and the subsequent information was reported in the ASTRE database.

The main study outcome was occurrence of a graft loss from 3-month post-KT to last updating in July 2020 or to patient death. Secondary outcomes were creatinine values and HLA antibodies at 1-year post transplant, as well as HLA antibodies and acute cell rejection at latest news.

### Statistical analysis

LCMM separating the population into homogeneous subgroups of individuals according to their creatinine trajectory was computed with the R “lcmm” package. Models with one to five classes were estimated and the number of classes was chosen by minimization of the Bayesian Information Criterion (BIC) and according to the size of identified subgroups.

Regarding the expected non-linearity of the trajectories, polynomial functions of time (2–5) were considered in the models. The models were adjusted for cold ischemia time, ECD, preemptive transplant and hemodialysis session completion, which were factors known to influence the dynamics of creatinine trajectories. Recipients were *a posteriori* classified in the class where they had the highest class-membership probability. The LCMM results are reported following the published “Guidelines for Reporting on Latent Trajectory Studies” (26).

We performed a sensitivity analysis excluding creatinine values recorded within the 24 h following a hemodialysis session.

Clinical characteristics were compared between the different latent classes identified by Chi2 or Fisher tests for qualitative variables and by ANOVA or Kruskal-Wallis test for quantitative variables. Dunn’s *post hoc* tests were performed in case of significance.

Association between latent classes and GL at one year and at the date of latest news was tested by a chi2 test. Time to GL was described by Kaplan-Meier curves in each latent class and compared using a logrank test. Adjustment for all factors found associated with GL was performed using a Cox model. Other qualitative outcomes were tested by a chi2 test and creatine values at one year were tested by ANOVA or Kruskal-Wallis test.

Statistical analyses were performed with R software (R Foundation for Statistical Computing, Vienna, Austria, version 4.0.3).

The results were considered significant for *p* values < 0.05.

## Results

**FIGURE 1:**
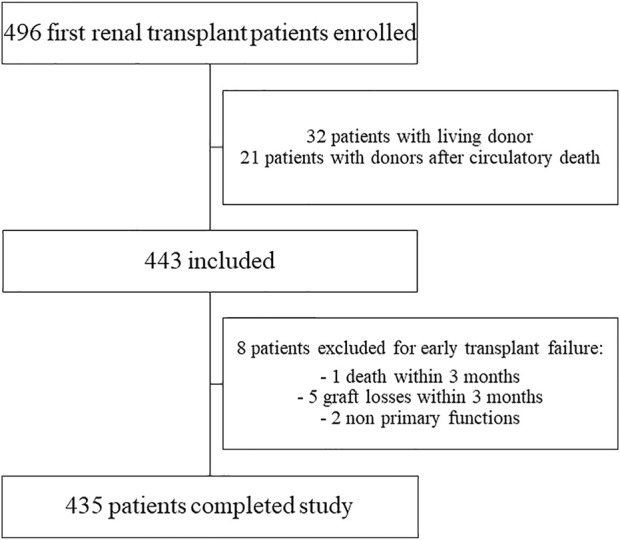
Flow chart of population selection from ASTRE cohort.

In the ASTRE cohort, 496 patients underwent a rank 1 KT at the Poitiers University Hospital. After applying the selection criteria, 435 recipients (258 males, 177 females aged 56 years) with kidney transplants from a DBD donor were included in the analysis (Figure 1).

Recipients, donors and transplants background characteristics are displayed in Table 1.

**TABLE 1:** Recipient, donor and transplant background characteristics in the overall population according to latent classes.

	All (*n* = 435)	Class 1 (*n* = 25) *poor recovery*	Class 2 (*n* = 206) *intermediate recovery*	Class 3 (*n* = 45) *good recover*y	Class 4 (*n* = 159) *optimal recovery*	*p*
Recipient characteristics						
Age (years)	56 (47–63)	58 (49–65)	56 (48–64)	57 (46–62)	56 (45–62)	0.3989
Male	258 (59%)	23 (92%)	129 (63%)	16 (36%)	90 (57%)	**<0.0001**
BMI, kg/m^2^	25 (22–28)	27 (24–31)	25 (23–29)	24 (20–28)	24 (21–27)	**0.0009**
CV disease	389 (89%)	24 (96%)	181 (88%)	38 (84%)	146 (92%)	0.2988
Hypertension	368 (85%)	22 (88%)	175 (85%)	36 (80%)	135 (85%)	0.8224
Diabetes	61 (14%)	2 (8%)	31 (15%)	7 (16%)	21 (13%)	0.8334
PRA status						**0.0459**
0 to 25	367 (84%)	22 (88%)	177 (66%)	31 (69%)	137 (86%)	
25 to 50	20 (5%)	2 (8%)	10 (5%)	2 (4%)	6 (4%)	
50 to 100	48 (11%)	1 (4%)	19 (9%)	12 (3%)	16 (10%)	
Preemptive transplantation	80 (18%)	3 (12%)	38 (18%)	4 (9%)	35 (22%)	0.2009
Waiting time on dialysis (month) (n = 355)	15 (5–29)	15 (9–30)	14 (4–28)	19 (10–34)	16 (2–29)	0.1005
Pre-operative hemodialysis	73 (16%)	7 (28%)	26 (13%)	14 (31%)	26 (16%)	**0.0109**
Donor characteristics						
Age (years)	55 (45–64)	56 (52–64)	56 (48–65)	54 (40–62)	55 (40–64)	0.1389
Male	255 (59%)	18 (72%)	116 (56%)	30 (67%)	91 (57%)	0.3049
BMI, kg/m^2^	25 (22–29)	25 (21–29)	25 (23–29)	25 (22–29)	25 (22–28)	0.6943
Expanded criteria donor	202 (46%)	12 (48%)	104 (50%)	16 (36%)	70 (44%)	0.2729
Hypertension	137 (31%)	7 (28%)	81 (39%)	11 (24%)	38 (24%)	**0.0082**
Diabetes	32 (7%)	3 (12%)	17 (8%)	2 (4%)	10 (6%)	0.5918
Transplantation characteristics						
Cold ischemia time (hours)	15 (12–18)	16 (13–18)	15 (13–18)	17 (13–22)	14 (12–17)	**0.0093**
Hypothermic machine perfusion	113 (26%)	5 (20%)	53 (26%)	7 (16%)	48 (30%)	0.2259
HLA-A mismatches						0.314
0	56 (13%)	3 (12%)	28 (14%)	10 (22%)	15 (9%)	
1	236 (54%)	16 (64%)	114 (55%)	21 (47%)	85 (53%)	
2	143 (33%)	6 (24%)	64 (31%)	14 (31%)	59 (37%)	
HLA-B mismatches						0.7986
0	24 (6%)	2 (8%)	9 (4%)	4 (9%)	9 (6%)	
1	201 (46%)	10 (40%)	97 (47%)	22 (49%)	72 (45%)	
2	210 (48%)	13 (52%)	100 (49%)	19 (42%)	78 (49%)	
HLA-DR mismatches						0.5899
0	106 (24%)	2 (7%)	50 (24%)	11 (25%)	43 (28%)	
1	240 (55%)	17 (63%)	116 (56%)	25 (57%)	82 (53%)	
2	89 (20%)	8 (30%)	42 (20%)	8 (18%)	31 (20%)	

Numbers are median (25th-75th percentiles) or effective (%); BMI, body mass index; PRA, panel reactive antibody.

The bold values are the significant values.

Between 24 h post-KT and hospital discharge, 6,211 serum creatinine measurements were recorded in the 435 patients corresponding to a median of 12 (11,12,13,14,15) measurements per patient per stay in agreement with median hospital stay duration. As shown in Table 2, delayed graft function was observed in 80 patients (18%) and a median number of 2 (2,3,4) hemodialysis sessions was needed during the hospital stay.

**TABLE 2:** Post-transplant characteristics in the overall population according to latent classes.

	All (*n* = 435)	Class 1 (*n* = 25) *poor recovery*	Class 2 (*n* = 206) *intermediate recovery*	Class 3 (*n* = 45) *good recover*y	Class 4 (*n* = 159) *optimal recovery*	*p*
Postgraft creatininemia at 24 h, mg/dL	6 (4–8)	9 (7–11)	6 (5–8)	5 (4–7)	6 (4–7)	**<0.0001**
Delayed graft function	80 (18%)	19 (76%)	43 (21%)	6 (13%)	12 (8%)	**<0.0001**
Nb. of hemodialysis sessions	2 (2–4)	4 (3–8)	2 (1–4)	2 (2–3)	2 (2–2)	**0.0057**
Time to reach the 2.83 mg/dL threshold (days)	3 (2–5)	33 (27–39)	4 (3–8)	3 (2–4)	3 (2–3)	**<0.0001**
Nadir creatinine	138 (103–202)	396 (302–450)	184 (140–237)	88 (71–106)	107 (90–135)	**<0.0001**
Patient’s hospital stay duration	12 (11–15)	20 (17–24)	13 (12–17)	13 (11–15)	11 (10–12)	**<0.0001**

Numbers are median (25th-75th percentiles) or effective (%); Delayed Graft Function: the need for a hemodialysis session in the first 7 days post-transplantation.

The bold values are the significant values.

During median follow-up of 73 (48–107) months, 68 patients (16%) lost their graft. At year one 7 GL (2%) and 39 T cell acute rejections (9%) were recorded.

### Subgroups of Serum Creatinine Trajectories

The choice of the various LCMM parameters are detailed in Supplementary Table S1. The best model, with the lowest BIC and a high entropy, determined 4 latent classes, whether there were adjustment variables or not (Supplementary Table S2). The mean posterior probabilities of class membership for individuals ranged from 81% to 87%, indicating good overall discrimination ability regarding the adjusted model.

**FIGURE 2:**
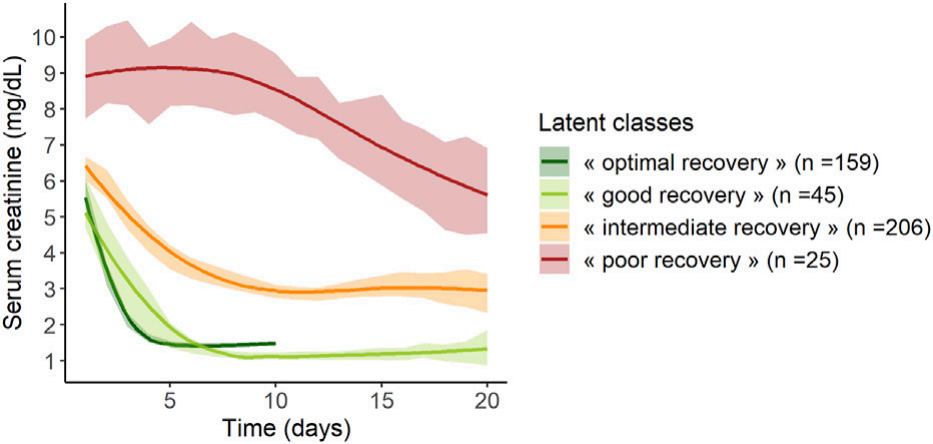
Mean trajectories of serum creatinine according to the 4 classes of the mixed latent class model (*n* = 435). Lines represent mean values and areas represent their 95% confidence intervals.

The mean trajectories of creatinine in the four latent classes are shown on Figure 2.

Class 1 (“*poor recovery*”) contained 25 patients (6%) with high values of serum creatinine at day 1, around 9 mg/dL, which decreased slowly over time, ending at 5.5 mg/dL at day 20 post KT. Class 2 (“*intermediate recovery*”) was the more numerous one with 206 patients (47%) showing moderate values of creatinine at day 1, around 6.5 mg/dL, which fell to 3 mg/dL within 10 days post-KT. Patients classified in Class 3 (“*good recovery*”, *n* = 45, 10%) and patients in Class 4 (“*optimal recovery*,” *n* = 159, 37%) had the lowest creatinine values at day 1, 5, and 5.5 mg/dL respectively; they both dropped to approximately 1.1–1.3 mg/dL at day 8 and day 4 respectively. After this pronounced diminution, “good recovery” patients’ creatinine remained stable, whereas “*optimal recovery*” patients’ creatinine showed a small rise of approximately 0.5 mg/dL from day 5 to day 10.

LCMM performed on the database excluding creatinine values reported within the 24 h following a hemodialysis session gave comparable results, identifying four classes with similar trajectories (*n* = 23 in Class 1, *n* = 204 in Class 2, *n* = 44 in Class 3 and *n* = 164 in Class 4).

### Characteristics of recipients and donors according to renal function trajectories

The characteristics of the recipients, donors and transplants according to the 4 latent classes are shown in Table 1. Recipients did not differ in age, history of hypertension, diabetes or immunization. Men and recipients with high body mass index (BMI) were more represented in the “*poor recovery*” class (92% men, median BMI value: 27 (24,25,26,27,28,29,30,31) kg/m^2^). Frequency of pre-emptive transplantation and waiting time on dialysis did not differ between classes.

Characteristics of donors did not differ between the four groups, including ECD criteria.

Regarding transplant characteristics, the median of cold ischemia time was different between the four classes (*p* = 0.0093); it was significantly lower in the “*optimal recovery*” group compared to the “good recovery” group (*p* = 0.0074).

Multivariate polynomial logistic regression showed that donor hypertension and cold ischemia time were the only independent predictors of latent classes with R-squared (R2) value not exceeding 6%.

In post-KT (Table 2), 24-hour creatinine values were significantly lower in “*optimal recovery*” and “good recovery” classes than in the others. DGF was more frequent and the number of hemodialysis sessions was higher in “*poor recovery*” recipients than in the others. Moreover, the time to reach the 2.83 mg/dL creatinine threshold and the nadir creatinine were significantly higher for “*poor recovery*” recipients. The latter had a significantly longer hospital stay than the others.

### Prognosis values of latent classes of creatinine

**FIGURE 3:**
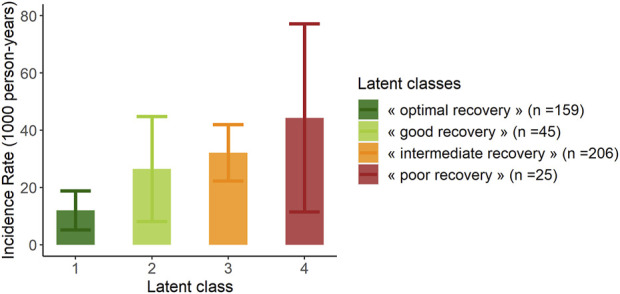
Incidence rate of graft loss at latest news according to the 4 classes of the mixed latent class model (*n* = 435). Error bars represent confidence intervals.

**FIGURE 4:**
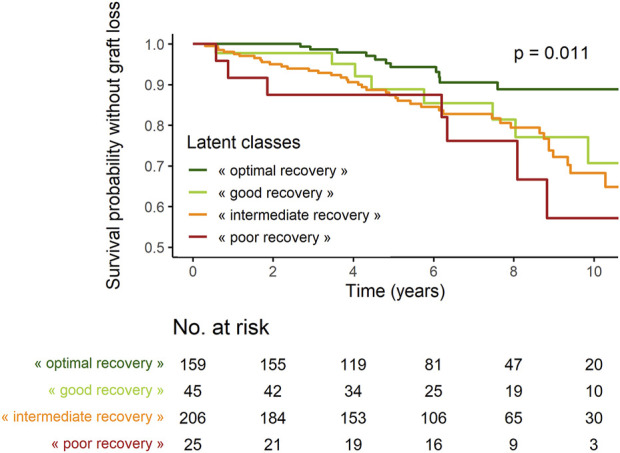
Survival probabilities without graft loss according to latent classes.

The proportion of recipients with GL during follow-up was significantly lower in “*optimal recovery*” patients (p_chi-deux_ = 0.0015) (Table 3). Considering time to event, incidence of GL was also lower in “*optimal recovery*” patients (p_logrank_ = 0.011) (Figures 3, 4). After adjustment for all factors found associated with GL in a univariate analysis (Supplementary Table S3), trajectories of creatinine were still significantly associated with GL: Compared with “*optimal recovery*” class, the risk of graft loss was 2.42 times higher for patients classified in “*intermediate recovery*” class and 4.06 times higher for patients classified in “*poor recovery*” class (Table 4). of note, nadir creatinine during hospital stay did not remain associated with GL when latent classes were considered in the model (*p* > 0.9).

**TABLE 3:** Graft outcomes in the overall population according to the different latent classes.

	All (n = 435)	Class 1 (*n* = 25) *poor recovery*	Class 2 (*n* = 206) *intermediate recovery*	Class 3 (*n* = 45) *good recover*y	Class 4 (*n* = 159) *optimal recovery*	*p*
Follow-up duration (months)	73 (48–107)	83 (49–106)	72 (48–107)	85 (48–118)	73 (48–98)	0.773
Graft loss, n (%)						
latest news	68 (16%)	7 (28%)	41 (20%)	8 (18%)	12 (8%)	**0.0015**
at year 1	7 (2%)	2 (8%)	4 (2%)	1 (2%)	0 (0%)	**0.0264**
Postgraft creatininemia at year 1, mg/dL (n = 428)*	1 (1–2)	2 (1–2)	2 (1–2)	1 (1–1)	1 (1–2)	**<0.0001**
T cell acute rejection at year 1, n (%)	39 (9%)	4 (16%)	18 (9%)	2 (4%)	15 (9%)	0.4478
Anti-HLA antibodies, n (%)						
at year 1 (n = 412)*	55 (13%)	4 (16%)	22 (11%)	6 (13%)	23 (14%)	0.5726
latest news (n = 415)*	76 (17%)	6 (24%)	37 (18%)	7 (16%)	26 (16%)	0.6626

Numbers are median (25th-75th percentiles) or effective (%); *7 patients lost their graft during the first year.

The bold values are the significant values.

**TABLE 4:** Predictive factors of graft loss: Cox regression model.

Characteristic	HR^a^	95% CI^a^	*p*
Pre-emptive graft			**0.028**
No	—	—	
Yes	0.43	0.18, 1.00	
BMI donor, kg/m^2^	1.05	1.01, 1.10	**0.014**
Expanded criteria donor			**<0.001**
No	—	—	
Yes	2.54	1.49, 4.35	
HLA-B mismatches			**0.033**
0	—	—	
1	0.29	0.12, 0.68	
2	0.41	0.18, 0.93	
Latent classes			**0.010**
« *optimal recovery* »	—	—	
« good recovery »	2.07	0.84, 5.11	
« *intermediate recovery* »	2.42	1.26, 4.63	
« *poor recovery* »	4.06	1.59, 10.4	

^a^ HR, adjusted Hazard Ratio; CI, confidence interval.

The bold values are the significant values.

Serum creatinine levels one year after KT were significantly different between the four classes with higher levels in “*poor recovery*” recipients (median of 2 (1,2) mg/dL) and lower levels in “*optimal recovery*” and “good recovery” recipients (medians of 1 (1) mg/dL and 1 (1,2) mg/dL respectively).

Biopsy-proven acute T cell rejection was found in 39 patients (9%) one year after KT and did not differ between the four latent classes.

Anti-HLA antibodies were not found significantly different between the latent classes neither at one year after KT nor at the latest news.

## Discussion

In the present study, using a retrospective cohort of 435 kidney transplant recipients, we performed a LCMM and highlighted substantial heterogeneity in renal function recovery immediately following KT from DBD. Interestingly, we identified four distinct trajectories showing non-linear decrease in daily creatinine levels during the initial hospitalization for transplantation.

These trajectories showed heterogeneity between patients regarding both creatinine level at day 1 and speed of the decrease, which reflects graft recovery. Patients with “optimal” or “good recovery” represented 37% and 10% of the study population, respectively, and their creatinine values dropped quickly. Patients with “*intermediate recovery*” had higher creatinine values at day one and a slower decrease over time, and they were the most numerous, representing 47% of the population. Finally, 6% of patients presented a trajectory markedly different from the others, with high initial values and a delayed and small decrease (“*poor recovery*”). The slight increase observed in the trajectory of the “*optimal recovery*” group could be due to the renal toxicity of the calcineurin inhibitors.

Although baseline characteristics were poor predictors of these trajectories, some clinically relevant variables such as cold ischemia time were associated with the trajectories. Interestingly, kidney donor hypertension, but not ECD, was also associated with the trajectories: in the “*optimal recovery*” trajectory, the lowest cold ischemia time and rate of hypertensive donors were found. The use of machines perfusion did not differ significantly between the different latent classes.

The four trajectories were also associated with relevant post-KT characteristics. As expected, patients classified in the “*poor recovery*” class were more likely to have DGF. Furthermore, they had the highest nadir creatinine and the longest time in days needed to reach the creatinine threshold of 2.83 mg/dL. “Good” and “*optimal recovery*” trajectories did not differ significantly in terms of time needed to reach the 2.83 mg/dL creatinine threshold.

Regarding graft outcome, the four trajectories were found to be an independent prognostic factor. In the literature, there are a few risk scores predicting graft loss, which include mainly donor and baseline transplant characteristics as prognostic factors (27,28). Cold ischemia time is identified as a risk factor for reduced graft and patient survival and DGF (29). The hypothermic perfusion machine also reduces the DGF incidence from 38% to 24% (30). Moreover, it is demonstrated that the survival of grafts at 1 year is 92.3% for grafts placed on a hypothermic machine perfusion, versus 80.2% for grafts with cold storage (31). During the first few months post-KT, 3-, 6- and 12-month eGFR have been reported as prognostic values (32) such as creatinine and eGFR trajectories beyond one year of follow-up (20,33). Nevertheless, none of these risk scores takes into consideration the evolution of daily creatinine levels in early post-KT. To our knowledge, our study is the first to characterize renal recovery trajectories immediately following KT, using the latent class analysis tool. Consideration of these daily determinations of creatinine during the hospital stay could be more relevant to predict GL than isolated values such as nadir of creatinine level.

The use of LCMM for the identification of trajectories of renal function evolution has raised a lot of interest in recent years (17,34). This novel unsupervised approach overlooks prior assumptions on the evolution of creatinine as a means of classifying patients. Our model was fitted on several covariates known to influence renal recovery, and this statistical fitting results in independency between these covariates and the identified trajectories. More specifically, the impact of hemodialysis sessions was taken into account as a time-dependent variable since it induces a drop in creatinine levels that gradually affects the creatinine levels, up to 24–48 h after the session. In a sensitivity analysis, we applied the model on the subset of data free of post-hemodialysis creatinine values and obtained four latent classes comparable to the previous ones.

Regarding the clinical applicability of our models, the type of recovery for a given recipient could be estimated outside the framework of this study. Indeed, the available hospital stay creatinine values and the covariates present in the model could be calculated in a dedicated application which would indicate the class membership probabilities. Hence, transplant recipients at higher risk of graft loss could be identified upon their hospital discharge, enabling personalized follow-up frequency and better management.

This study has several limitations, particularly its monocentric design. Selection bias regarding recipients and donors might have conditioned class-membership. External validation is needed to confirm the general applicability of a LCMM. Moreover, an analysis conducted on Maastricht-III and living donors could be of particular interest.

In conclusion, substantial inter-individual heterogeneity in creatinine trajectories immediately following KT was highlighted. Insight into a recovery class might lead to more precise estimation of risk of graft loss for a given patient and be conducive to optimized post-KT management.

## Data Availability

The original contributions presented in the study are included in the article/Supplementary Material, further inquiries can be directed to the corresponding author.

## Abbreviations

BIC, Bayesian Information Criterion; BMI, Body Mass Index; CKD, Chronic Kidney Disease; DBD, Death after Brain Death; DGF, Delayed Graft Function; ECD, Expanded Criteria Donor; ESRD, End-Stage Renal Disease; GFR, Glomerular Filtration Rate; GL, Graft Loss; KT, Kidney Transplantation; LCMM, Latent Class Mixed Model.
