# Supplementary material for: Paving the Way for Personalized Medicine in First Kidney Transplantation: Interest of a Creatininemia Latent Class Analysis in Early Post-transplantation
Source: Transpl Int. 2023 Feb 16;36:10685. doi: 10.3389/ti.2023.10685 (PMC9977818; doi:10.3389/ti.2023.10685)
Supplement: Supplementary file 1 [file DataSheet1.docx]

# Supplementary table 1: Choice of the mixed latent class model regarding random effects (a), link function (b) and number of splines (c).

## 1.a : Choice of random effects

| Random effects | Convergence | BIC | Log-likelihood | Entropy |
| --- | --- | --- | --- | --- |
| t | Yes | 73940 | -36952 | 1 |
| **t+t^2^** | **Yes** | **71380** | **-35660** | **1** |
| t+t^2^+t^3^ | No | 41011640 | -20505774 | 1 |
| t+t^2^+t^3^+t^4^ | No | 84194 | -42033 | 1 |
| t+t^2^+t^3^+t^4^+t^5^ | No | 2000000170 | -1000000000 | 1 |

## 1.b : Choice of link function

| Link function | Convergence | BIC | Log-likelihood | Entropy |
| --- | --- | --- | --- | --- |
| Linear | Yes | 73940 | -36952 | 1 |
| Bêta | Yes | 65980 | -32953 | 1 |
| **Splines** | **Yes** | **65589** | **-32749** | **1** |

## 1.c : Choice of number of splines

| No. of nodes | Convergence | BIC | Log-likelihood | Entropy |
| --- | --- | --- | --- | --- |
| 2 at quanti | Yes | 68340 | -34134 | 1 |
| 3 at quanti | Yes | 65344 | -32632 | 1 |
| 4 at quanti | Yes | 65273 | -32594 | 1 |
| **5 at quanti** | **Yes** | **65247** | **-32578** | **1** |
| 6 at quanti | Yes | 65247 | -32578 | 1 |
| 7 at quanti | Yes | 65247 | -32578 | 1 |
| 8 at quanti | Yes | 65247 | -32578 | 1 |
| 9 at quanti | Yes | 65247 | -32578 | 1 |

###

# Supplementary table 2: Choice of the number of classes and posterior classification table without the adjustment variables (a) and with the adjustment variables (b).

## 2.a. posterior classification table without the adjustment variables

|  | | | Mean of posterior probabilities | | | | |  | |
| --- | --- | --- | --- | --- | --- | --- | --- | --- | --- |
| No. of classes | BIC | Class 1 | | Class 2 | Class 3 | Class 4 | Class 5 | | Entropy |
| None | 65247 | 1.0000 | |  |  |  |  | | 1.0000000 |
| Two | 65242 | 0.9188 | | 0.8088 |  |  |  | | 0.6261899 |
| Three | 65247 | 0.8502 | | 0.9448 | 0.9185 |  |  | | 0.8402902 |
| **Four** | **65232** | **0.8234** | | **0.8644** | **0.9356** | **0.8181** |  | | **0.7232571** |
| Five | 65246 | 0.8161 | | 0.7301 | 0.8663 | 0.9192 | 0.7288 | | 0.6946197 |

## 2.b. posterior classification table with the adjustment variables

|  | | | Mean of posterior probabilities | | | | |  | |
| --- | --- | --- | --- | --- | --- | --- | --- | --- | --- |
| No. of classes | BIC | Class 1 | | Class 2 | Class 3 | Class 4 | Class 5 | | Entropy |
| None | 65228 | 1.0000 | |  |  |  |  | | 1.0000000 |
| Two | 65235 | 0.9528 | | 0.7724 |  |  |  | | 0.7622660 |
| Three | 65231 | 0.7617 | | 0.8537 | 0.7891 |  |  | | 0.6480187 |
| **Four** | **65218** | **0.8682** | | **0.8551** | **0.8654** | **0.8143** |  | | **0.7182414** |
| Five | 65232 | 0.8571 | | 0.7251 | 0.8509 | 0.8897 | 0.7311 | | 0.6876907 |

# Supplementary table 3: Characteristics of recipients, donors and transplantation according to graft loss.

|  | Yes  (n=68) | No  (n=367) | p |
| --- | --- | --- | --- |
| Recipient characteristics |  |  |  |
| Age (years) | 59 (49-66) | 56 (47-62) | **0.0371** |
| Male | 43 (63%) | 215 (59%) | 0.4732 |
| BMI, kg/m² | 25 (23-28) | 25 (22-28) | 0.2729 |
| CV disease | 59 (87%) | 330 (90%) | 0.4373 |
| Hypertension | 55 (81%) | 313 (85%) | 0.3555 |
| Diabetes | 11 (16%) | 50 (14%) | 0.5777 |
| PRA status |  |  | 0.9999 |
| 0 to 25 (n=367) | 58 (85%) | 309 (84%) |  |
| 25 to 50 (n=20) | 3 (4%) | 17 (5%) |  |
| 50 to 100 (n=48) | 7 (10%) | 41 (11%) |  |
| Pre-emptive transplantation | 6 (9%) | 74 (20%) | **0.0266** |
| Waiting time on dialysis (month) (n=355) | 18 (10-32) | 15 (3-29) | **0.0375** |
| Donor characteristics |  |  |  |
| Age (years) | 60 (52-66) | 55 (44-64) | **0.0061** |
| Male | 42 (62%) | 213 (58%) | 0.5666 |
| BMI, kg/m² | 27 (23-33) | 25 (22-28) | **0.0020** |
| Extended criteria donor | 45 (66%) | 157 (43%) | **0.0004** |
| Hypertension | 37 (54%) | 100 (27%) | **<0.0001** |
| Diabetes | 7 (10%) | 25 (7%) | 0.3221 |
| Transplantation characteristics |  |  |  |
| Cold ischemia time (hours) | 16 (13-20) | 15 (12-17) | **0.034** |
| Hypothermic machine perfusion | 16 (24%) | 97 (26%) | 0.6446 |
| HLA-A mismatches |  |  | 0.0810 |
| 0 | 5 (7%) | 51 (14%) |  |
| 1 | 45 (66%) | 191 (52%) |  |
| 2 | 18 (26%) | 125 (34%) |  |
| HLA-B mismatches |  |  | **0.0295** |
| 0 | 7 (10%) | 17 (5%) |  |
| 1 | 23 (34%) | 178 (49%) |  |
| 2 | 38 (56%) | 172 (47%) |  |
| HLA-DR mismatches |  |  | 0.0685 |
| 0 | 10 (15%) | 96 (26%) |  |
| 1 | 39 (57%) | 201 (55%) |  |
| 2 | 19 (28%) | 70 (19%) |  |
| Post-transplantation characteristics |  |  |  |
| Postgraft creatininemia at 24 hours, mg/dL | 6 (4-8) | 6 (4-8) | 0.6298 |
| Delayed graft function | 24 (35%) | 56 (15%) | **0.0001** |
| Nb. of hemodialysis sessions | 2 (2-4) | 2 (2-4) | 0.4682 |
| Time to reach 2.83 mg/dL (days) | 3 (3-6) | 3 (2-5) | 0.0745 |
| Nadir creatinine, mg/dL | 2 (1-3) | 2 (1-2) | **0.0055** |
| Hospital stay duration (days) | 13 (12-17) | 12 (11-14) | **0.0001** |
| Latent classes |  |  | **0.0015** |
| « poor recovery » | 7 (10%) | 18 (5%) |  |
| « intermediate recovery » | 41 (60%) | 165 (45%) |  |
| « good recovery » | 8 (12%) | 37 (10%) |  |
| « optimal recovery » | 12 (18%) | 147 (40%) |  |
| Graft outcomes |  |  |  |
| Postgraft creatininemia at year 1, mg/dL | 2 (1-2) | 1 (1-2) | **<0.0001** |
| T-cell acute rejection at year 1 | 16 (24%) | 23 (6%) | **<0.0001** |
| Anti-HLA antibodies |  |  |  |
| at year 1 (n=415) | 16 (24%) | 36 (10%) | **<0.0001** |
| latest news | 19 (28%) | 49 (13%) | **<0.0001** |
